# Hierarchical Recognition Scheme for Human Facial Expression Recognition Systems

**DOI:** 10.3390/s131216682

**Published:** 2013-12-05

**Authors:** Muhammad Hameed Siddiqi, Sungyoung Lee, Young-Koo Lee, Adil Mehmood Khan, Phan Tran Ho Truc

**Affiliations:** 1 UC Lab, Department of Computer Engineering, Kyung Hee University, Yongin-Si 446-701, Korea; E-Mails: siddiqi@oslab.khu.ac.kr (M.H.S.); sylee@oslab.khu.ac.kr (S.L.); yklee@khu.ac.kr (Y.-K.L.); 2 Division of Information and Computer Engineering, Ajou University, Suwon 443-749, Korea; E-Mail: amtareen@ajou.ac.kr

**Keywords:** face detection, GHE, facial expressions, PCA, ICA, LDA, HMMs

## Abstract

Over the last decade, human facial expressions recognition (FER) has emerged as an important research area. Several factors make FER a challenging research problem. These include varying light conditions in training and test images; need for automatic and accurate face detection before feature extraction; and high similarity among different expressions that makes it difficult to distinguish these expressions with a high accuracy. This work implements a hierarchical linear discriminant analysis-based facial expressions recognition (HL-FER) system to tackle these problems. Unlike the previous systems, the HL-FER uses a pre-processing step to eliminate light effects, incorporates a new automatic face detection scheme, employs methods to extract both global and local features, and utilizes a HL-FER to overcome the problem of high similarity among different expressions. Unlike most of the previous works that were evaluated using a single dataset, the performance of the HL-FER is assessed using three publicly available datasets under three different experimental settings: n-fold cross validation based on subjects for each dataset separately; n-fold cross validation rule based on datasets; and, finally, a last set of experiments to assess the effectiveness of each module of the HL-FER separately. Weighted average recognition accuracy of 98.7% across three different datasets, using three classifiers, indicates the success of employing the HL-FER for human FER.

## 1. Introduction

Over the last decade automatic FER has become an important research area for many applications, such as more engaging human–computer interfaces; image retrieval; human emotion analysis [1]; neuroscience, psychology, and cognitive sciences [2]; access control and surveillance [3]; and communication, personality, and child development [4].

Human FER systems can be classified into two categories; pose-based expression recognition systems [5–7] and spontaneous expression recognition systems [8–10]. Pose-based expressions are the artificial expressions produced by people when they are asked to do so [2]. Similarly, spontaneous expressions are those that people give out spontaneously, and they can be observed on a day-to-day basis, such as during conservations or while watching movies [2]. The focus of this article is pose-based FER systems.

**Figure 1.**
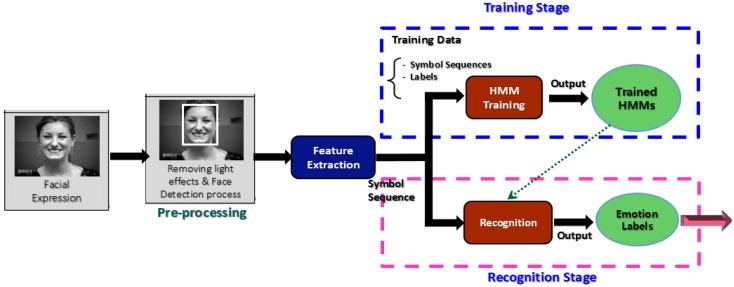
Typical architecture of general facial expression recognition systems.

As shown in Figure 1, a typical FER system consists of three basic modules: pre-processing, feature extraction, and recognition. The pre-processing module performs two tasks. Firstly, it diminishes illumination and other light effects to increase the recognition accuracy, using techniques like morphological filters, homomorphic filters, or median filters. Most previously FER systems [11–15] have exhibited low accuracy due to the lack of this filtering element in their architecture. One of the most well-known methods that have been utilized to diminish such illumination effects is histogram equalization (HE). However, in a gray level image HE assigns the highest gray level value to each pixel [16], as a result the resulting image produced by HE contains gaps *i.e.*, “empty-bins” between very full histogram bins [17]. Due to this limitation, HE causes unwanted artifacts and a washed-out look, so it is not recommended [18,19].

Therefore, two improved versions of HE were proposed in order to solve the limitations of HE. The first technique is Local Histogram Equalization (LHE) that uses a sliding window technique in order to compute the local histogram for each pixel and the gray level for center pixel is changed accordingly [17]. LHE, though better than HE, causes over-enhancement, and sometimes it produces checkerboards of the enhanced image [20] and it requires more time than other methods [21]. The second technique is Global Histogram Equalization (GHE), which uses the histogram information of the whole image; therefore, we employed GHE instead of using HE and LHE in preprocessing module. To the best of our knowledge, it is the first time that GHE is being used for facial expression recognition.

Naturally, before recognizing facial expressions a face is must be located in the image/video frame. Therefore, the second task of the preprocessing module is to detect faces in a given image, as it is the face that contains most of the expression-related information. For this, many well-known methods, including the appearance-based methods [22,23], have been proposed. The appearance-based methods have shown excellent performance in a static environment; however, their performance degrades when used in dynamic situations [24]. Other commonly used face detection methods include neural network-based face detection [25], and digital curves-based face detection [26]. These methods utilize eye window (eye regions) in order to locate a face. However, eyes regions are highly sensitive to the hairstyle of the person, which may cause misclassification [27]. Moreover, in their methods, it is very hard to know the orientation of the detected face [27]. Many existing works, such as [28–30], have performed facial expression recognition without face detection, making these systems heuristic in nature. To solve this problem, some face detection and extraction techniques were proposed [31–33]. In [31], the authors detected the human face using the position of the eyes and then cropped the faces accordingly. However, this method failed to detect the face structure in very sensitive environmental conditions. In contrast, in [33] features for face detection were selected manually.

PCA has been exploited by [34,35] to extract the features from eyes and the lips, which were then employed by a framework of radial basis function network (RBFN) to classify the expressions based on the extracted features. PCA extracts only the global features; therefore, another well-known higher statistical order technique named ICA has also been widely employed in FER systems to extract the local features. The authors of [36] employed supervised ICA for feature extraction in their FER system and showed improved recognition accuracies. As ICA is an unsupervised technique; therefore, in their work, they modified the classical ICA and made it a supervised ICA (sICA) by including a prior knowledge that was reassembled from the training data using a Maximum Posteriori (MAP) scheme. Other important feature extraction technique used for the sake of FER in the past include: non-negative matrix factorization (NMF) and Local Non-negative Matrix Factorization (LNNF) [37], Higher order Local Auto-Correlation (HLAC) [38], Local Binary Pattern (LBP) [13], Gabor wavelet [39], and BoostedICA [40].

Regarding the feature extraction module, several methods have been explored. Some of the most well-known features used for FER include texture-based features [41], geometry-based features [42], holistic features such as nearest features using line-based subspace analysis [43], Eigenfaces and Eigenvector [44–46], Fisherfaces [47], global features [48], and appearance based features [49,50]. In this article, we utilized the holistic feature extraction methods, *i.e.*, Principal Component Analysis (PCA) and Independent Component Analysis (ICA) in order to extract the prominent features from the expression frames.

As for the recognition module, several classifiers have been investigated. The authors of [51] employed artificial neural networks (ANNs) to recognize different types of facial expressions and achieved an accuracy of 70%. However, an ANN is a black box and has incomplete capability to explicitly categorize possible fundamental relationships [52]. Moreover, the FER systems proposed in [8,53–57] used support vector machines (SVMs). However, in SVMs, the probability is calculated using indirect techniques; in other words, there is no direct estimation of the probability, these are calculated by employing five-fold cross validation due to which SVM suffers from the lack of classification [58]. Furthermore, SVMs simply disregard the temporal addictions among video frames, thus each frame is expected to be statistically independent from the rest. Similarly, in [49,59,60], Gaussian mixture models (GMMs) were employed to recognize different types of facial expressions. As stated earlier, the features could be very sensitive to noise; therefore, fast variations in the facial frames cannot be modeled by GMMs and produces problems for sensitive detection [61]. Hidden Markov Models (HMMs) are mostly used to handle sequential data when frame-level features are used. In such cases, other vector-based classifiers like GMMs, ANNs, and SVMs, have difficulty in learning the dependencies in a given sequence of frames. Due to this capability, some well-known FER systems, including [62–64], utilized HMM as a classifier. In conclusion, a large number of feature extraction techniques and classifiers have been employed for video-based FER systems. Among them, PCA and ICA have been the most widely used feature extraction techniques, and HMMs have been the most commonly used classifier.

A recent work by Zia *et al.* [64] proposed a complete approach for FER systems that provided high classification accuracy for the Cohn-Kanade database of facial expressions. In their work, they employed PCA and ICA for feature extraction. Once extracted, features were subject to Linear Discriminant Analysis (LDA) to find the most relevant features. The result after applying LDA was fed to an HMM. The recognition rate of their technique was 93.23% when tested on Cohn-Kanade dataset. However, their technique failed in exhibiting the same accuracy when tested by us on other datasets, such as Japanese Female Facial Expression (JAFFE) database (83%), and AT&T database (72%) of facial expressions. Low accuracy in these new experiments could be attributed to the following two reasons. Firstly, in their work, they did not use a pre-processing step to diminish the lighting and illumination effects. Furthermore, in some of the databases such as the AT&T database of facial expressions, the subjects have worn glasses that make it difficult to extract useful features from some parts of the face, such as the eyes.

**Figure 2.**
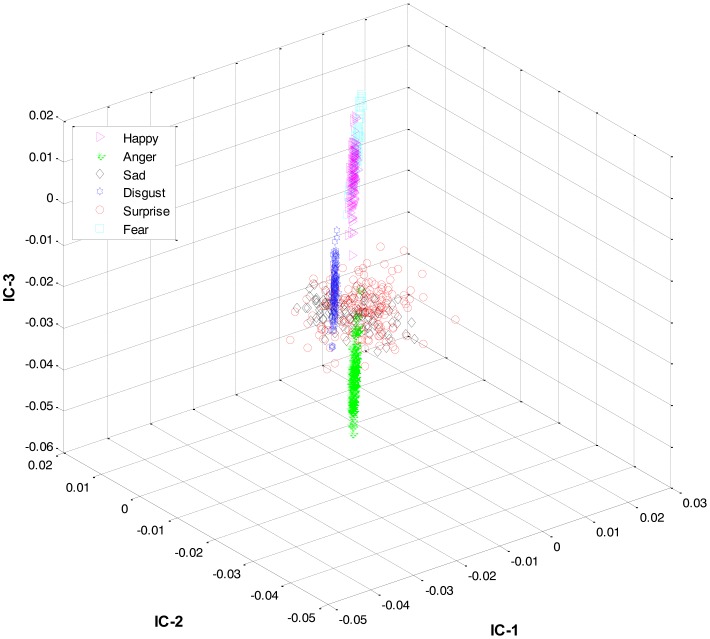
3D-feature plot of Cohn-Kanade dataset for six different types of facial expressions, where each expression has twelve expression frames. It can be seen that the features are highly merged, due to the presence of similarity between the expressions, which could later results in a high misclassification rate.

Secondly, most of the expressions share high similarity, and thus their features overlap significantly in the feature space, as shown in Figure 2. Zia *et al.* [64] applied LDA to the extracted feature space to improve the class separation among different classes with the assumption that the variance is distributed uniformly among all the classes. However, this is not the case. For example, expressions like happiness and sadness are very similar to each other but can easily be distinguished from anger and fear (another pair with high similarity). Accordingly, this work implements a HL-FER that is capable of performing accurate facial expression recognition across multiple datasets. Previously, such model has been used in [65] that was dual-layer SVM ensemble classification. The motivation behind their study was to determine how the contraction of muscles changes the appearance of the face by extracting the local features from the three parts of the face such as mouth, nose, and eyes. However, the performance of the dual-layer SVM classification cannot match that of binary classification as SVMs use approximation algorithms in order to decrease the computation complexity but these have the effect of degrading classification performance [66].

In our HL-FER, firstly, images were passed through a pre-processing module to diminish the illumination effects, and to extract the human face automatically. Secondly, PCA and ICA were used for feature extraction. Finally, a hierarchical classifier was used, where the expression category was recognized at the first level, followed by the actual expression recognition at the second level. The HL-FER has been validated using three different experiments. The results of these experiments show that the two-level recognition scheme, along with the proposed pre-processing module for noise reduction and accurate face detection, solved the aforementioned problems of the existing FER systems; and therefore, succeeded in providing high recognition accuracy across multiple datasets.

Above, we discussed some related work in this field. The rest of the paper is organized as follows. Section 2 provides an overview of our HL-FER. Section 3 discusses the experimental setup along with the experimental results with some discussion on the results and talks about the factors that could degrade systems performance if tested in real-life scenarios. Section 4 provides the analysis and comparison of the recognition accuracy of this work with those of the some of the existing FER systems. Finally, the paper is concluded with some future directions in Section 5.

## 2. Materials and Methods

**Figure 3.**
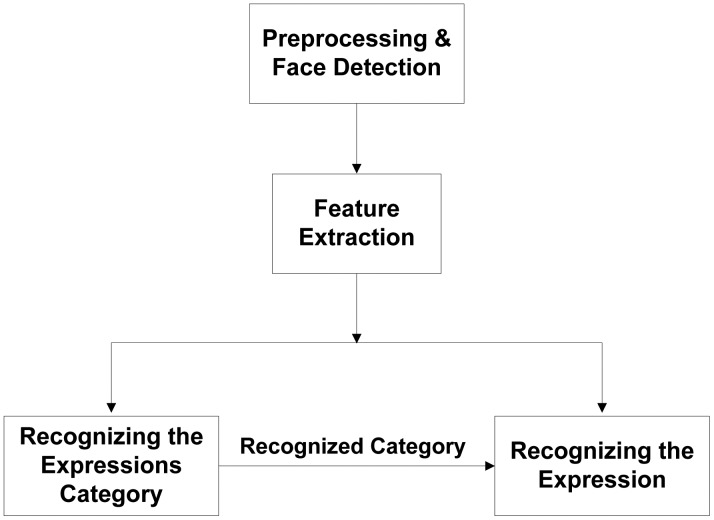
Architectural diagram for the HL-FER.

The architecture of our HL-FER is shown in Figure 3.

### 2.1. Pre-Processing

As mentioned earlier, in most of the datasets, the face images have various resolution and backgrounds, and were taken under varying light conditions; therefore, pre-processing module is necessary to improve the quality of images. At this stage, background information, illumination noise, and unnecessary details are removed to enable fast and easy processing. After employing this stage, we can obtain sequences of images which have normalized intensity, size and shape. Several techniques exist in literature to diminish such illumination effects, such methods are local histogram equalization (LHE) and global histogram equalization (GHE). However, LHE causes over-enhancement, and sometimes it produces checkerboards of the enhanced image [38]. Therefore, we employed a GHE for diminishing illumination effects. To the best of our knowledge, this is the first time GHE has been exploited for facial expression recognition. For more detail on GHE, please refer to [39].

In the proposed face detection algorithm, two improved key face detection methods were used simultaneously: gray-level and skin-tone-based. To attain the best performance, the similarity angle measurement (SAM) method was used. SAM utilizes two energy functions, *F*(*C*) and *B*(*C*), to minimize the dissimilarities within the face and maximize the distance between the face and the background, respectively. The overall energy function can be defined as:

(1) E(C)=βF(C)+(1−β)B(C)

Where *β* ∈ [0−1], and

(2) $$F\left(C\right)=\int_{\text{inside}(C)}{\left|I-c_{in}\right|}^{2}dx+\int_{\text{outside}(C)}{\left|I-c_{\text{out}}\right|}^{2}dx$$

where *c_in_* and *c_out_* are the average intensities inside and outside the variable boundary *C*, and *I* is the facial image. Furthermore:

(3) $$B\left(C\right)=\int_{z}\sqrt{f_{1}\left(x\right)f_{2}\left(x\right)}dx$$

where:

(4) $$f_{1}\left(x\right)=\frac{K_{\sigma }{\left(x\right)}^{*}\left[H\left(\phi \left(x\right)\right)I\right]}{K_{\sigma }{\left(x\right)}^{*}\left[H\left(\phi \left(x\right)\right)\right]}$$

and:

(5) $$f_{2}\left(x\right)=\frac{K_{\sigma }{\left(x\right)}^{*}\left[H\left(\phi \left(x\right)\right)I\right]}{K_{\sigma }{\left(x\right)}^{*}\left[1-H\left(\phi \left(x\right)\right)\right]}$$

where *f*_1_(*x*) and *f*_2_(*x*) are the local fitting functions, which depend on the facial geometry function *ϕ*(*x*), and need to be updated in each iteration, *x* is the corresponding region, and *H*(•) and *K_σ_*(•) as the Heaviside and Dirac functions, respectively.

In summary, the intuition behind the proposed energy functional for SAM is that we seek for a boundary which partitions the facial image into regions such that the differences within each region are minimized (*i.e.*, the *F*(*C*) term) and the distance between the two regions (face and background) is maximized (*i.e.*, the *B*(*C*) term). The facial geometry function *ϕ*(*x*) implementation for the energy functional in Equations (4) and (5) is carried out in order to define the boundary between the two regions (face and background) and can be derived as:

(6) $$\phi \left(x\right)=\frac{\partial \phi }{\partial t}=\left|\nabla \phi \right|\left\{\begin{array}{l} \gamma K_{\sigma }+\eta +\beta \left[{\left(I-c_{in}\right)}^{2}+{\left(I-c_{\text{out}}\right)}^{2}\right]\\ -\left(1-\beta \right)\left[\frac{B\left(C\right)}{2}\left(\frac{1}{A_{in}}-\frac{1}{A_{\text{out}}}\right)+\frac{1}{2}\int_{z}K_{\sigma }\left(z-1\right)\left(\frac{1}{A_{\text{out}}}\sqrt{\frac{f_{1}\left(x\right)}{f_{2}\left(x\right)}}-\frac{1}{A_{in}}\sqrt{\frac{f_{1}\left(x\right)}{f_{2}\left(x\right)}}\right)dz\right] \end{array}\right\}$$

Where |∇*_ϕ_*| is the gradient for the facial geometry function *ϕ*(*x*), and *A_in_* and *A_ou_*_t_ are respectively the areas inside and outside of the boundary *C*, *γ*, *β* and *η* are the three parameters, such that *γ* helps to detect objects of various sizes, including small points caused by noise; *β* weights the constraints of within-face homogeneity and between-face dissimilarity for which the value of *β* should be small; *η* speeds up the function evolution but may make it to pass through weak edges. Therefore, we used *η* = 0 in all experiments for fair comparison. Once the boundary between face and background is found, then in the next step, the skin-tone and gray-level methods are collectively applied in order to accurately detect and extract the face from the facial frame. For further details on gray-level and skin-tone methods please refer to [27,67], respectively.

In summary, the proposed face detection system is based on multiple features from a face image/frame. When a rough face image is presented to the system, an improved gray-level and skin-tone model is adopted to locate the face region. Very often, hair is included in the detected head contour. The second step is to find the precise face geometry using a facial geometry operation. To locate the exact face region, three features were used: the face intensity (because the intensity of the eye region is relatively low [27]); the direction of the line connecting the center of the eyes, which is determined on the face edge image; and the response of convolving the proposed facial geometry variance filter with the face image. We have developed a facial geometry filter for extracting potential face windows, based on similarity measurements of facial geometry signatures. This process generates a list of possible face signatures, since each face feature has unique identification, or signatures. These signatures can be compared with face signatures that are stored in the database to determine positive matches. The stored face signatures were in true (original) form and were not distorted. However, in some cases, the detected face boundary usually might consist of hair [68]. Hairstyles are different from person to person [69]. Therefore, in the next step another technique, *i.e.*, face skin region has been employed in order to locate the skin region from the detected face region and to get rid of the problem of different hairstyles.

**Figure 4.**
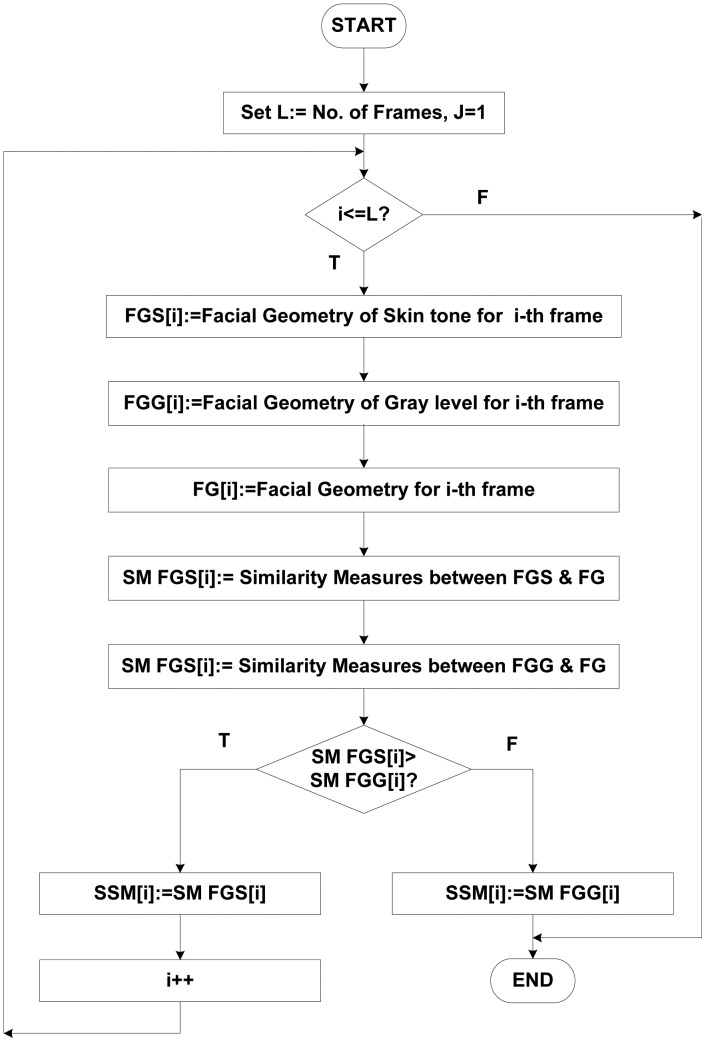
The control flow diagram for the proposed face detection system.

The facial features such as nose, mouth, and eyes possess relatively lower gray levels under normal illumination [27]. We can always plot the intensity histogram of the face image, because skin color has a relatively high gray intensity, while other facial components have relatively low intensities, and by this way, it is easy to find a threshold value for face detection under normal illumination. This threshold value, which is obtained by the intensity histogram, is used as the criterion for successful face detection. In most images, the number of possible cases for the second iteration of the loop (see Figure 4) was less than two. For each possible case, the signature similarity measurement (SSM) function was adopted for face detection, tracking, and verification. If the face was detected in the face frame, the detection process completed; if not, next possible case was tested. Details of each block are presented in Figure 4.

### 2.2. Feature Extraction

As described earlier, numerous techniques have been developed and validated for the purpose of feature extraction for FER systems. Among these, PCA and ICA are the mostly commonly used methods, and their performance has already been validated in [64]. Therefore, we decided to use PCA and ICA for feature extraction to extract both the global and local features respectively.

### 2.3. Recognizing the Expression Category

This work is based on the theory that different expressions can be grouped into three categories based on the parts of the face that contribute most toward the expression [70–72]. This classification is shown Table 1.

Lips-based expressions are those in which the lips make up the majority of the expression. In lips-eyes-based expressions, both lips and eyes contribute in the expression. In lips-eyes-forehead expressions, lips, eyes, and eyebrows or forehead have equal roles. In the HL-FER, an expression is classified into one of these three categories at the first level. At the second level, classifier (trained for the recognized category) is employed to give a label to this expression within this category.

**Figure 5.**
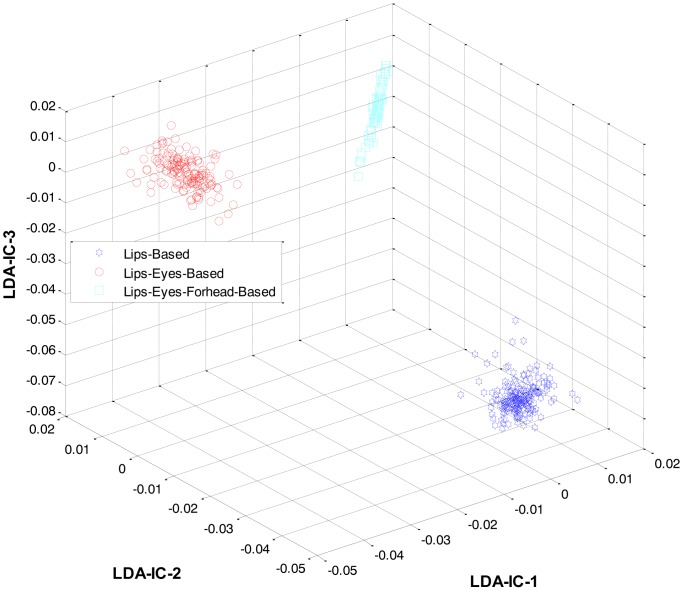
3D feature plots of the HL-FER after applying LDA at the first level for the three expression-categories such as lips-based, lips-eyes-based, or lips-eyes-forehead-based expressions (on Cohn-Kanade dataset). It can be seen that at the first level, the HL-FER achieved 100% classification rate in expressions categories classification.

At the first level, LDA was firstly applied to the extracted features from all the classes and an HMM was trained to recognize the three expression categories: lips-based, lips-eyes-based, or lips-eyes-forehead-based expressions. The LDA-features for these three categories are shown in Figure 5. A clear separation could be seen among the categories, and this is why the HL-FER achieved 100% recognition accuracy at the first level.

### 2.4. Recognizing the Expressions

**Figure 6.**
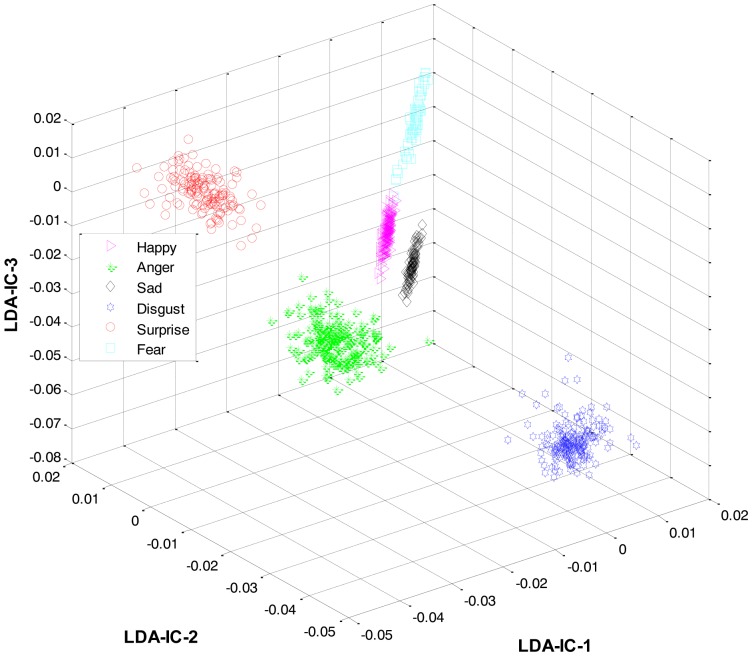
3D feature plots of the HL-FER after applying LDA at the second level for recognizing the expressions in each category (on Cohn-Kanade dataset). It can be seen that at the second level, the HL-FER achieved much higher recognition rate as compared to a single-LDA via single-HMM shown in Figure 7.

As mentioned earlier, once the category of the given expression has been determined, the label for the expression within the recognized category is recognized at the second level. For this purpose, LDA was applied separately to the feature space of each category and the result was used to train three HMMs, one HMM per category. Collectively, the overall results for all the expression classes are shown in Figure 6.

**Figure 7.**
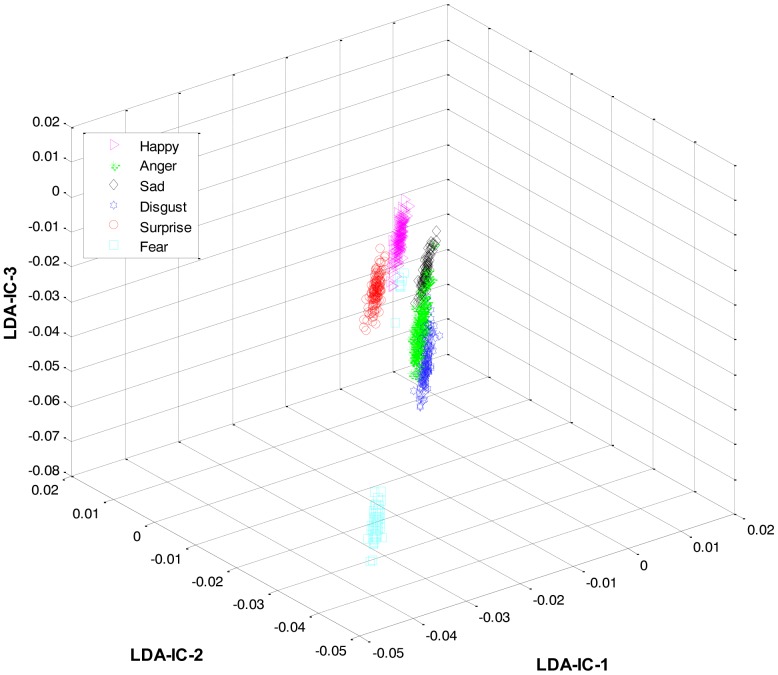
3D-feature plot of single-LDA via single-HMM (on Cohn-Kanade dataset). It can be seen that using a single-LDA via single-HMM approach did not yield as good a separation among different classes as was achieved by the HL-FER (See Figure 6).

**Figure 8.**
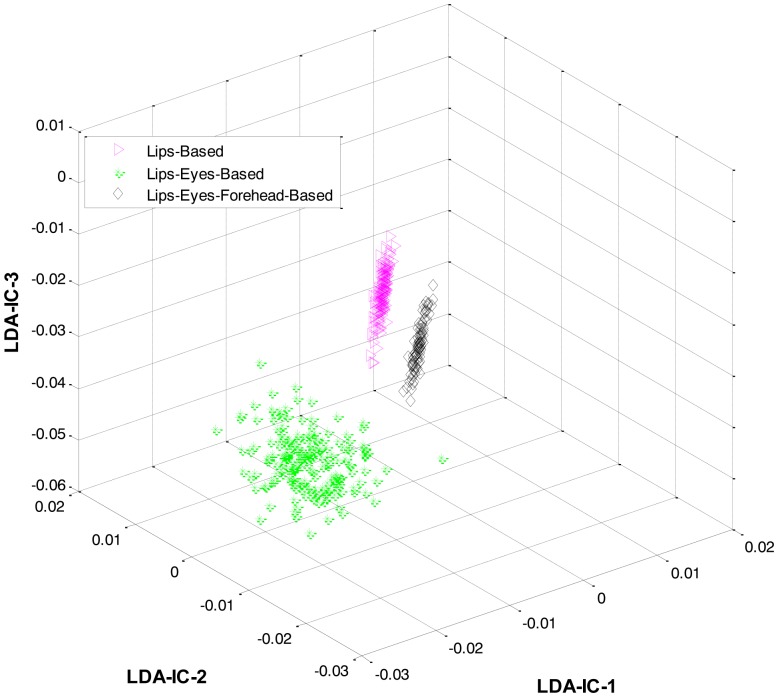
3D feature plots of the HL-FER after applying LDA at the first level for the three expression-categories such as lips-based, lips-eyes-based, or lips-eyes-forehead-based expressions (on JAFFE dataset). It can be seen that at the first level, the HL-FER achieved 100% classification rate in expressions categories classification.

**Figure 9.**
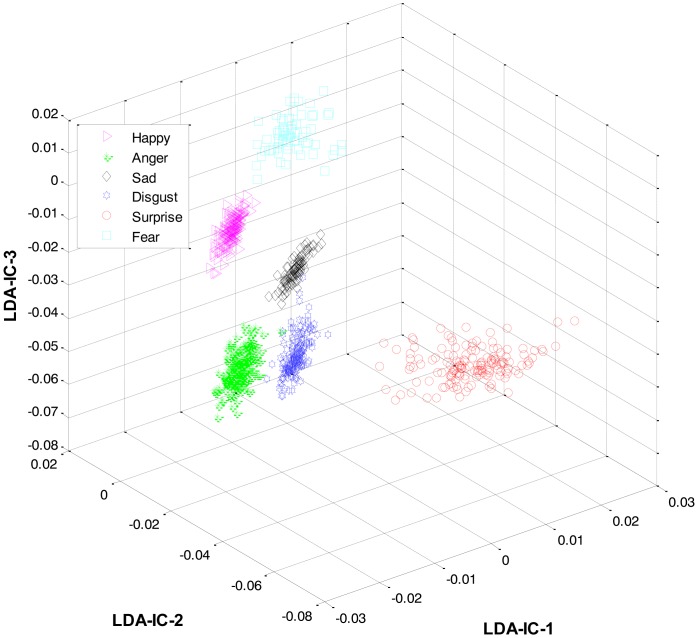
3D feature plots of the HL-FER after applying LDA at the second level for recognizing the expressions in each category (on JAFFE dataset). It can be seen that at the second level, the HL-FER achieved much higher recognition rate as compared to a single-LDA via single-HMM shown in Figure 10.

**Figure 10.**
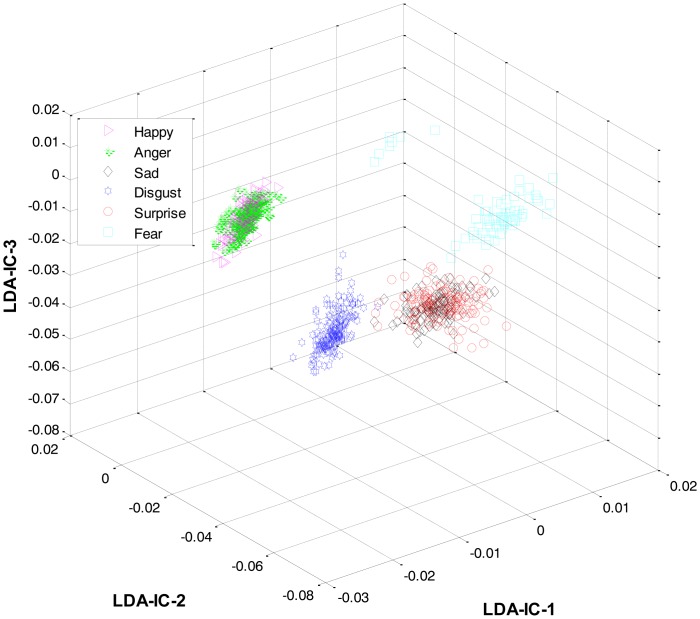
3D-feature plot of single-LDA via single-HMM (on JAFFE dataset). It can be seen that using a single-LDA via single-HMM approach did not yield as good a separation among different classes as was achieved by the HL-FER (See Figure 9).

**Figure 11.**
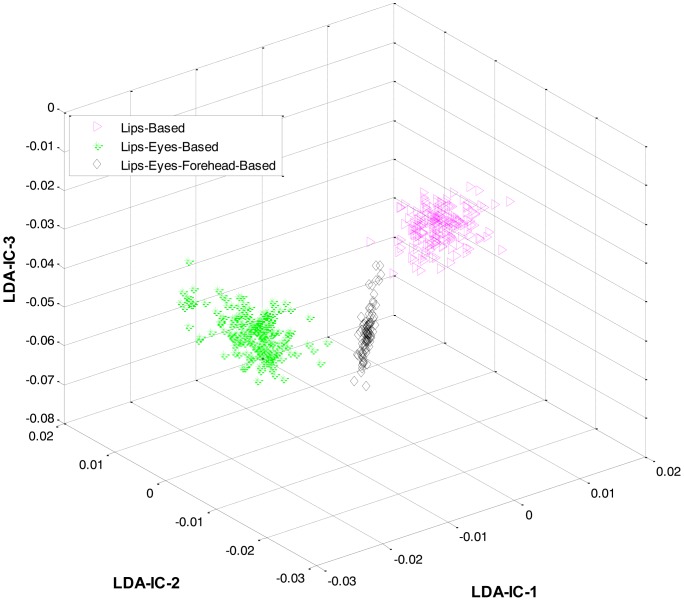
3D feature plots of the HL-FER after applying LDA at the first level for the three expression-categories such as lips-based, lips-eyes-based, or lips-eyes-forehead-based expressions (on AT&T dataset). It can be seen that at the first level, the HL-FER achieved 100% classification rate in expressions categories classification.

**Figure 12.**
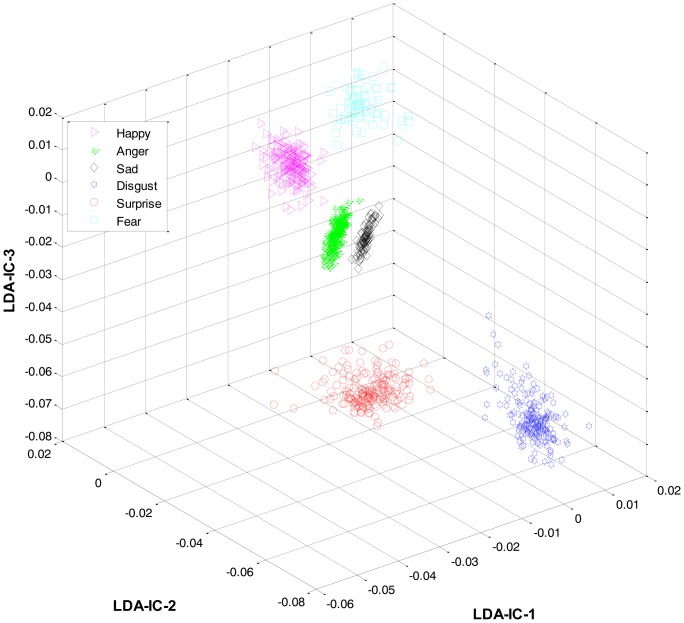
3D feature plots of the HL-FER after applying LDA at the second level for recognizing the expressions in each category (on AT&T dataset). It can be seen that at the second level, the HL-FER achieved much higher recognition rate as compared to a single-LDA via single-HMM shown in Figure 13.

**Figure 13.**
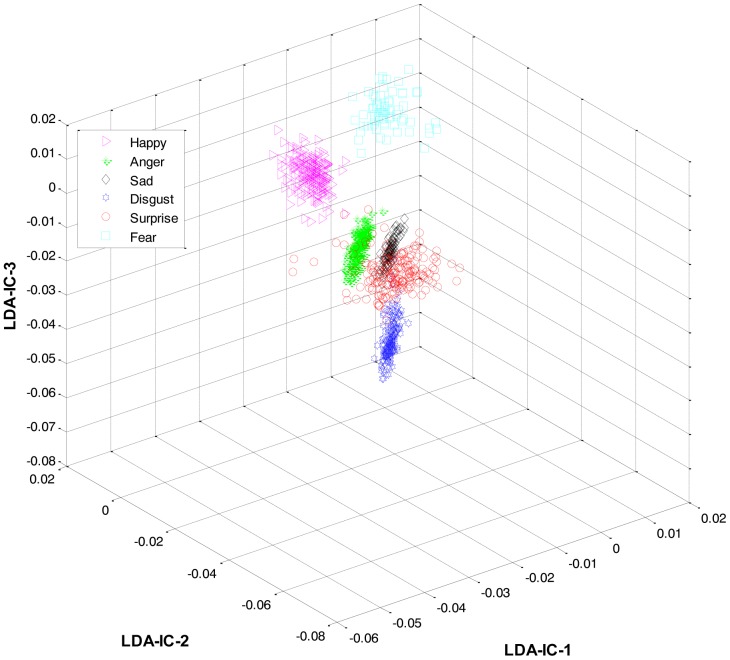
3D-feature plot of single-LDA via single-HMM (on AT&T dataset). It can be seen that using a single-LDA via single-HMM approach did not yield as good a separation among different classes as was achieved by the HL-FER (See Figure 12).

These feature plots indicate that applying LDA to the features of three categories separately provided a much better separation as compared to single-LDA via single-HMM approach (see Figure 7). The single-LDA via single-HMM approach means that instead of applying LDA separately to each expression category and using separate HMMs for these categories, LDA is applied only once, to the features of all the classes, and only one HMM is used for classification. For Figures 5, 6 and 7, we used Cohn-Kanade dataset, for Figures 8, 9 and 10, we employed JAFFE dataset, and for Figures 11, 12 and 13, we used AT&T dataset. Each dataset consisted of six basic universal facial expressions and each expression has twelve facial expression frames.

We have sequential data at both levels; therefore at both levels HHMs have been employed, because HMMs have their own advantage of handling sequential data when frame-level features are used. In such cases, other vector-based classifiers like GMMs, ANNs, and SVMs, have difficulty in learning the dependencies in a given sequence of frames. The following formula that has been utilized in order to model HMM (*λ*):

(7) λ=(O,Q,π)

where *O* is the sequence of observations e.g., *O*_1_, *O*_2_,…, *O_T_* and each state is denoted by *Q* such as *Q* = *q*_1_, *q*_2_,…, *q*_N_, where *N* is the number of the states in the model, and *π* is the initial state probabilities. The parameters that used to model HMM for all experiments were 44, 4, and 4 respectively.

## 3. Experimental Results

### 3.1. Setup

In order to assess the HL-FER, six universal expressions like: happiness, sadness, surprise, disgust, anger, and fear were used from three publicly available standard datasets, namely the Cohn-Kanade [73], JAFFE [74] and AT&T [75] datasets. These datasets display the frontal view of the face, and each expression is composed of several sequences of expression frames. During each experiment, we reduced the size of each input image (expression frame) to 60 × 60, where the images were fi wh converted to a zero-mean vector of size 1 × 3,600 for feature extraction. All the experiments were performed in Matlab using a dual-core Pentium processor (2.5 GHz) with a RAM capacity of 3 GB. Some information on the three datasets is as follows:

#### 3.1.1. Cohn-Kanade Dataset

In this facial expressions dataset, there were 100 subjects (university students) performed basic six expressions. The age range of the subjects were from 18 to 30 years and most of them were female. We employed those expression for which the camera was fixed in front of the subjects. By the given instructions, the subjects performed a series of 23 facial displays. Six expressions were based on descriptions of prototypic emotions such as happy, anger, sad, surprise, disgust, and fear. In order to utilize these six expressions from this dataset, we employed total 450 image sequences from 100 subjects, and each of them was considered as one of the six basic universal expressions. The size of each facial frame was 640 × 480 or 640 × 490 pixel with 8-bit precision for grayscale values. For recognition purpose, twelve expression frames were taken from each expression sequence, which resulted in a total of 5,400 expression images.

#### 3.1.2. JAFFE Dataset

We also employed Japanese Female Facial Expressions (JAFFE) dataset in order to assess the performance of the HL-FER. The expressions in the dataset were posed by 10 different (Japanese female) subjects. Each image has been rated on six expression adjectives by 60 Japanese subjects. Most of the expression frames were taken from the frontal view of the camera with tied hair in order to expose all the sensitive regions of the face. In the whole dataset, there were total 213 facial frames, which consists of seven expressions including neutral. Therefore, we selected 205 expression frames for six facial expressions performed by ten different Japanese female as subjects. The size of each facial frame was 256 × 256 pixels.

#### 3.1.3. AT&T Dataset

Additionally, we also employed AT&T dataset of facial expressions to evaluate the performance of the HL-FER. There are 10 facial frames in each expression performed by 40 distinct subjects. The frames were taken at different illuminations of light against a dark homogenous background with the subjects, and were in grey scale having size of 92 × 112 pixels. This dataset consists of open/close eyes, smiling and not smiling expressions. Among these expressions, few expressions that showed the basic six expressions; therefore, we have manually chosen those suitable facial frames for our experiments. The total numbers of selected facial frames were 240.

**Figure A1.**
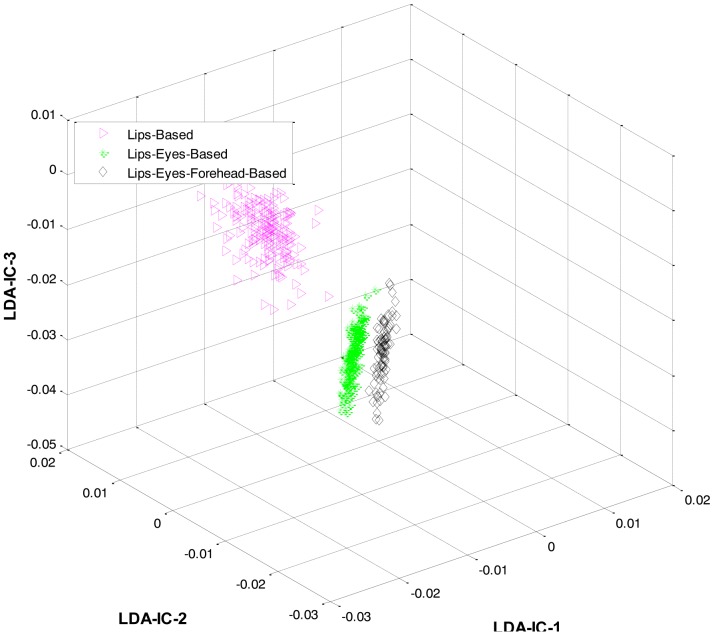
3D feature plots of the HL-FER after applying LDA at the first level for the three expression-categories such as lips-based, lips-eyes-based, or lips-eyes-forehead-based expressions on Yale B dataset for six different types of facial expressions. It can be seen that at the first level, the HL-FER achieved 100% classification rate in expressions categories classification.

**Figure A2.**
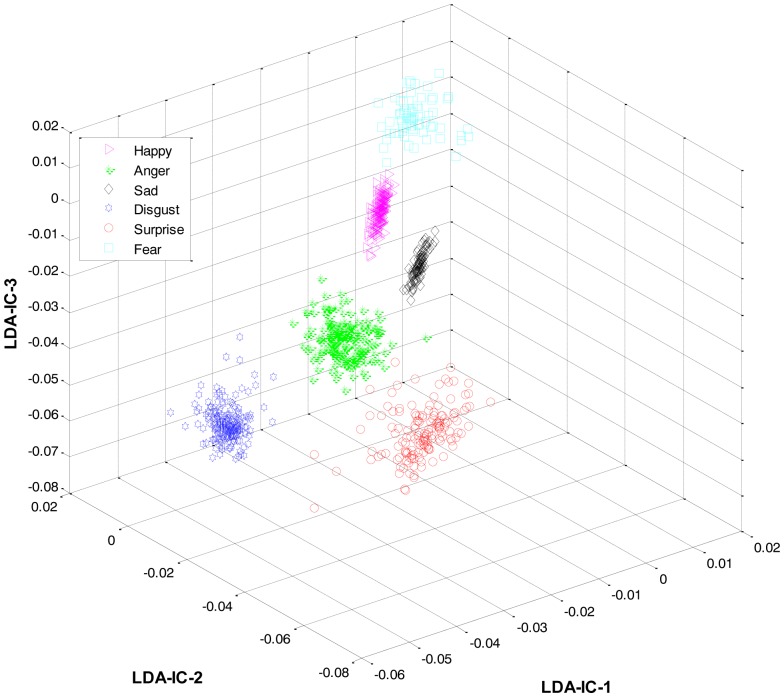
3D feature plots of the HL-FER after applying LDA at the second level for recognizing the expressions in each category on Yale B dataset for six different types of facial expressions. It can be seen that at the second level, the HL-FER achieved much higher recognition rate as compared to a single-LDA via single-HMM shown in Figure 7.

**Figure A3.**
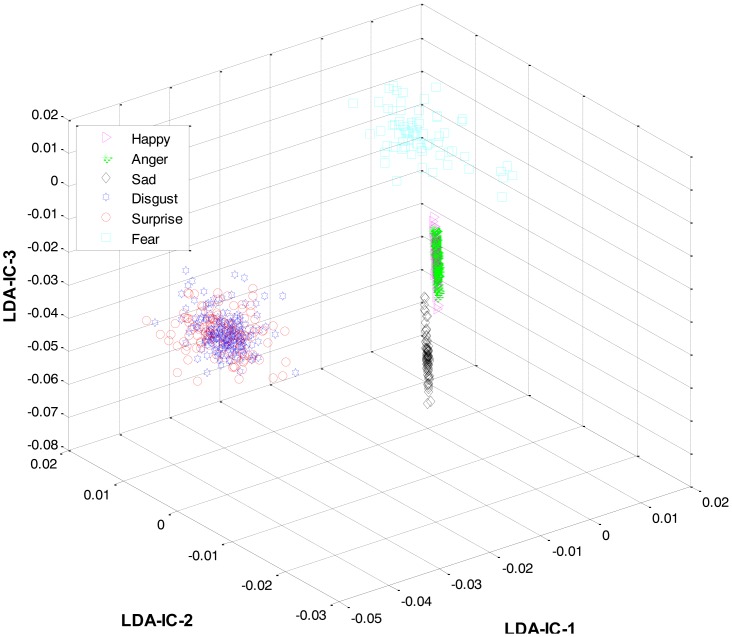
3D-feature plot of single-LDA via single-HMM on Yale B dataset for six different types of facial expressions. It can be seen that using a single-LDA via single-HMM approach did not yield as good a separation among different classes as was achieved by the HL-FER (See Figure 6).

Moreover, in order to show the efficacy of the HL-FER, we performed some more experiments on Yale database of facial expressions [76]. The results are shown in the Appendix in Figures A1, A2, A3, and A4 and in Table A5 respectively.

### 3.2. Results and Discussion

#### 3.2.1. Experimental Face Detection Method Results

**Figure 14.**
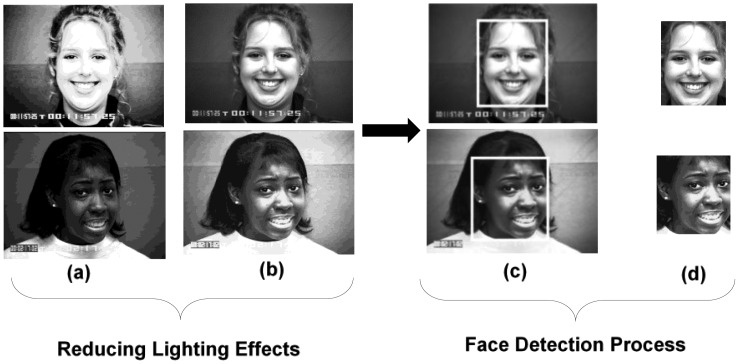
Sample results for the proposed GHE along with the face detection method.

Some samples results for the proposed preprocessing method (GHE) along with the proposed face detection are shown in Figure 14. Figure 14b indicates the success of the proposed GHE in diminishing the lighting effects from both a bright and dark image (shown in Figure 14a) in order to enhance the facial features. After this, in the next step, the proposed face detection method found the facial faces in the frames (shown in Figure 14c) and cropped it accordingly, as shown in Figure 14d.

The detection rates of the proposed face detection algorithm for the three datasets are summarized in Table 2. It can be seen from Table 2 that the proposed face detection algorithm achieved high recognition rate on the three different standard datasets of facial expressions.

#### 3.2.2. Experimental Results of HL-FER Based on Subjects

The experiments for the HL-FER were performed in this order. In the fin t experiment, the HL-FER was validated on three different datasets. Each dataset possessed different facial features, such as some of the subjects have worn glasses in AT&T dataset while the subjects of the Cohn-Kanade and JAFFE datasets are free of glasses. The remaining facial features of AT&T and Cohn-Kanade datasets are quite similar with each other. On the other hand, the facial features, such as the eyes of the subjects in the JAFFE dataset are totally different from the eyes of the subjects of AT&T and Cohn-Kanade datasets [77,78].The HL-FER was evaluated for each dataset separately that means for each dataset, *n*-fold cross-validation rule (based on subjects) was applied. It means that out of *n* subjects, data from a single subject was retained as the validation data for testing the HL-FER, whereas the data for the remaining *n* − 1 subjects were used as the training data. This process was repeated *n* times, with data from each subject used exactly once as the validation data. The value of *n* varied according to the dataset used. The detailed results of this experiment for the three datasets are shown in Tables 3, 4 and 5, respectively.

**Figure 15.**
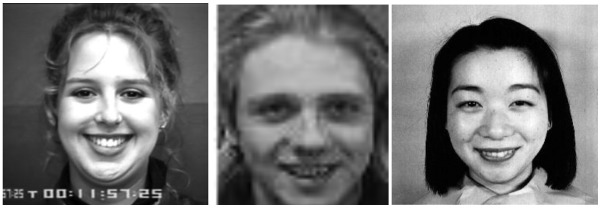
Sample reference images for three datasets: from left to right: Cohn-Kanade dataset, AT&T dataset, and JAFFE dataset, respectively.

Despite of all these differences, the HL-FER consistently achieved a high recognition rate when applied on these datasets separately, *i.e.*, 98.87% on Cohn-Kanade, 98.80% on JAFFE, and 98.50% on the AT&T dataset. This means that, unlike Zia *et al.* [64], the HL-FER is robust *i.e.*, the system not only achieves high recognition rate on one dataset but shows the same performance on other datasets as well. The reference images of the three datasets are shown in Figure 15.

#### 3.2.3. Experimental Results of HL-FER Based on Datasets

In the second experiment the HL-FER cross-dataset validation was performed. This means that from the three datasets, data from the two datasets were retained as the validation data for testing the system, and the data from the remaining dataset was used as the training data. This process was repeated three times, with data from each dataset used exactly once as the training data.

The experimental results of the HL-FER at the first level classification for the category recognition on Cohn-Kanade dataset is shown in Table 6, while on the JAFFE dataset, the results are indicated in Table 7, and for the AT&T dataset, the results are represented in Table 8. Similarly, the experimental results of the HL-FER at the second level are summarized in Tables 9, 10, and 11 respectively.

#### 3.2.4. Experimental Results of HL-FER under the Absence of Each Module

Thirdly, a set of experiments was performed to assess the effectiveness of each module of the HL-FER (pre-processing, face detection and hierarchical recognition) separately. This experiment was repeated three times and the recognition performance was analyzed under three different settings: Firstly, the experiment was repeated without the pre-processing step. Secondly, the experiment was performed without including the face detection module. In this case the accuracy for the HL-FER is same as indicated in Tables 3, 4 and 5, *i.e.*, when the proposed face detection method fails to detect the face in the facial frame, then there is no effect on the accuracy of the HL-FER, but the HL-FER processes the whole frame instead processing the region of interest (face); however, it will take a bit more time to recognize the expression by considering the whole frame. And, lastly, a single LDA and HMM were used to recognize all the expressions instead of using the HL-FER. The results for the three settings on the Cohn-Kanade dataset are shown in Tables 12, 3 and 13, on the JAFFE dataset are presented in Tables 14, 4, and 15, and on the AT&T dataset are displayed in Tables 16, 5, and 17, respectively.

It can be seen from Tables 12, 13, 14, 15, 16 and 17 that both the preprocessing and hierarchical recognition modules of the HL-FER are important. As indicated in Tables 13, 15, and 17, the hierarchical recognition is mainly responsible for the high recognition accuracy of the HL-FER. When we removed the hierarchical recognition module, the recognition rate decreased significantly. These results support the theory that the problem of high similarity among the features of different expressions is a local problem. In other words, the features exist in the form of groups in the overall feature space. The expressions within one group are very similar, whereas they are easily distinguishable from those in the other groups; therefore, to overcome this problem in an effective manner, these groups (or expression categories) should be separated first and then techniques like LDA should be applied to each category separately.

In the next experiment, the accuracy of the HL-FER when tested across different datasets decreased significantly. We believe that this decrease in accuracy is due to training the HL-FER using the JAFFE dataset and then testing the HL-FER on the AT&T or Cohn-Kanade datasets, and *vice versa*. As explained earlier, the facial structures, especially eyes, of the subjects in the Japanese dataset are very different than those of the AT&T and Cohn-Kanade datasets [79], which acts as noise and thus degrades the HL-FER performance. To test this theory, another experiment was performed where the HL-FER was first trained using the Cohn-Kanade and then tested on the AT&T dataset, and then the same experiment was repeated while switching the datasets. The results of these experiments are shown in Tables 18 and 19.

It can be seen from the Tables 18 and 19 that the HL-FER achieved good results and proved the early stated theory of lower recognition accuracy due to the difference in the facial features of the subjects in the JAFFE dataset and the other two datasets.

## 4. Comparison and Analysis of HL-FER

The performance of the HL-FER was compared against nine conventional methods [34–40,64,79], for all the three datasets, *i.e.*, the Cohn-Kanade, JAFFE, and AT&T datasets of facial expressions. All of these methods were implemented by us using the instructions provided in their respective papers. For each dataset, *n*-fold cross-validation rule (based on subjects) was applied. In other words, out of *n* subjects, data from a single subject was retained as the validation data for testing the HL-FER, whereas the data for the remaining *n* − 1 subjects were used as the training data. This process was repeated *n* times, where the value of *n* varied according to the dataset used. The average recognition results (for the three datasets) for each of these conventional methods are listed in Table 20. It can be seen that the HL-FER outperformed the existing facial expression systems.

Lastly, to analyze the computational cost of the HL-FER, its average recognition time for the three datasets was compared to that of [64], as [64] achieved the highest recognition accuracy in the above experiment among the nine conventional methods. The average computational time taken by [64] for the Cohn-Kanade, JAFFE, and AT&T datasets was 662, 375, and 590 ms, respectively. The datasets had 5,400, 205, and 240 images, respectively. On the other hand, the HL-FER took 1,655 ms for the Cohn-Kanade dataset, 639 ms for the JAFFE dataset, and 1,180 ms for the AT&T dataset. These results show that though the HL-FER showed significant improvement over conventional methods in terms of recognition accuracy, it achieved that at the expense of a higher computational cost. In the HL-FER, we used two level classifications and in each level, we utilized HMM for classification; therefore, the HL-FER took a bit more time than of the existing method [64].

All these experiments were performed in the laboratory (offline validation) using three standard datasets. Though the system performed accurately and robustly in all the experiments, the effects on system performance once implemented in real time are yet to be investigated. There exist several elements that could degrade the systems performance, such as the clutter and varying face angles. Clutter means that there could be some unnecessary objects in the images along with the test subject. Solving such problems would require a real-time robust segmentation technique. Moreover, the images used in this work were taken only from the frontal view; however, in real life the angles of the camera might vary *i.e.*, side views can also happen. Therefore, further study is needed to tackle these issues and maintain the same high recognition rate in real-life environment.

Eventually, we envision that the HL-FER will be employed in smartphones. Even though our system showed high accuracy, it employs two-level-recognition with HMMs used at each level. This might become a complexity issue, especially when used in smartphones. One solution could be to use a lightweight classifier such as k-nearest neighbor (k-NN) at the first level; however k-NN has its own limitations such as it is very sensitive to the presence of inappropriate parameters and sensitive to noise as well. Therefore, it can have poor performance in a real-time environment if the training set is large. Therefore, further study is needed to find ways to maintain the high accuracy of the HL-FER while improving its efficiency at the same time to be used in smartphones.

## 5. Conclusions

Over the last decade, automatic human FER has become an important research area for many applications. Several factors make FER a challenging research problem, such as varying light conditions in training and test images, the need for automatic and accurate detection of faces before feature extraction, and the high similarity among different expressions resulting in overlaps among feature values of different classes in the feature space. That is why, though several FER systems have been proposed that showed promising results for a certain dataset, their performance was significantly reduced when tested with different datasets. In this paper, we proposed and validated the accuracy of a HL-FER. Unlike the previous systems, the HL-FER involved a pre-processing step based on global histogram equalization (GHE), which helped in provide high accuracy by eliminating the light effects. We also proposed a face detection technique that utilizes both gray levels and skin-tones to automatically detect faces with high accuracy. Further, we employed both PCA and ICA to extract both the global and the local features. Finally, we used a HL-FER to overcome the problem of high similarity among different expressions. Expressions were divided into three categories based on different parts of the face. At the first level, LDA was used with an HMM to recognize the expression category. At the second level, the label for an expression within the recognized category is recognized using a separate set of LDA and HMM, trained just for that category. In order to evaluate the performance of the HL-FER detailed experiments were conducted on three publicly available datasets in three different experimental settings. The HL-FER achieved a weighted average recognition accuracy of 98.7% using three HMMs, one for per category expression across three different datasets (the Cohn-Kanade dataset has 5,400 images, the JAFFE dataset has 205 images, and the AT&T dataset has 240 images), illustrating the successful use of the HL-FER for automatic FER.

## Figures and Tables

**Table 1.** The classified categories and facial expressions recognized in this study.

**Category**	**Facial Expressions**
**Lips-Based**	**Happiness** **Sadness**
**Lips-Eyes-Based**	**Surprise** **Disgust**
**Lips-Eyes-Forehead-Based**	**Anger** **Fear**

**Table 2.** Average accuracy rates of the proposed face detection algorithm on three publically available datasets (Unit: %).

**Datasets**	**Detection Rate**
Cohn-Kanade	99
JAFFE	99
AT&T	98
Average	98.7

**Table 3.** Confusion matrix for the HL-FER using the Cohn-Kanade database of facial expressions (Unit: %).

	**Happiness**	**Sadness**	**Anger**	**Disgust**	**Surprise**	**Fear**
**Happiness**	**98**	2	0	0	0	0
**Sadness**	2	**98**	0	0	0	0
**Anger**	0	0	**99**	0	0	1
**Disgust**	0	0	0	**99**	1	0
**Surprise**	0	0	0	0	**100**	0
**Fear**	0	0	2	0	0	**98**
**Average**	**98.87**					

**Table 4.** Confusion matrix for the HL-FER using the JAFFE database of facial expressions (Unit: %).

	**Happiness**	**Sadness**	**Anger**	**Disgust**	**Surprise**	**Fear**
**Happiness**	**99**	1	0	0	0	0
**Sadness**	2	**98**	0	0	0	0
**Anger**	0	0	**100**	0	0	0
**Disgust**	0	0	0	**99**	1	0
**Surprise**	0	0	0	3	**97**	0
**Fear**	0	0	0	0	0	**100**
**Average**	**98.80**					

**Table 5.** Confusion matrix for the HL-FER using the AT&T database of facial expressions (Unit: %).

	**Happiness**	**Sadness**	**Anger**	**Disgust**	**Surprise**	**Fear**
**Happiness**	**98**	2	0	0	0	0
**Sadness**	1	**99**	0	0	0	0
**Anger**	0	0	**99**	0	0	1
**Disgust**	0	0	0	**98**	2	0
**Surprise**	0	0	0	2	**98**	0
**Fear**	0	0	1	0	0	**99**
**Average**	**98.50**					

**Table 6.** The recognition rate of the HL-FER at the first level for recognizing the expressions category such as Lips-Based, Lips-Eyes-Based, and Lips-Eyes-Forehead-Based on Cohn-Kanade dataset (Unit: %).

**Expressions Category**	**Recognition Rate**
Lips-Based	86
Lips-Eyes-Based	83
Lips-Eyes-Forehead-Based	87
Average	85.3

**Table 7.** The recognition rate of the HL-FER at the first level for recognizing the expressions category such as Lips-Based, Lips-Eyes-Based, and Lips-Eyes-Forehead-Based on the JAFFE dataset (Unit: %).

**Expressions Category**	**Recognition Rate**
Lips-Based	80
Lips-Eyes-Based	79
Lips-Eyes-Forehead-Based	82
Average	80.3

**Table 8.** The recognition rate of the HL-FER at the first level for recognizing the expressions category such as Lips-Based, Lips-Eyes-Based, and Lips-Eyes-Forehead-Based on the AT&T dataset (Unit: %).

**Expressions Category**	**Recognition Rate**
Lips-Based	87
Lips-Eyes-Based	84
Lips-Eyes-Forehead-Based	82
Average	84.3

**Table 9.** Confusion matrix for the HL-FER, showing the weighted average recognition accuracy for six expressions. Training on Cohn-Kanade dataset and testing on the JAFFE and AT&T datasets (Unit: %).

	**Happiness**	**Sadness**	**Anger**	**Disgust**	**Surprise**	**Fear**
**Happiness**	**81**	4	3	4	2	6
**Sadness**	5	**85**	2	3	1	4
**Anger**	2	3	**82**	5	3	5
**Disgust**	5	4	3	**75**	7	6
**Surprise**	2	3	4	6	**80**	5
**Fear**	2	4	3	1	5	**85**
**Average**	**81.30**					

**Table 10.** Confusion matrix for the HL-FER, showing the weighted average recognition accuracy for six expressions. Training on the JAFFE dataset and testing on the Cohn-Kanade and AT&T datasets (Unit: %).

	**Happiness**	**Sadness**	**Anger**	**Disgust**	**Surprise**	**Fear**
**Happiness**	**71**	4	7	6	5	7
**Sadness**	8	**74**	6	5	3	4
**Anger**	4	6	**79**	5	3	3
**Disgust**	3	2	4	**81**	7	3
**Surprise**	5	5	4	2	**80**	4
**Fear**	3	5	8	2	6	**76**
**Average**	**76.80**					

**Table 11.** Confusion matrix for the HL-FER, showing the weighted average recognition accuracy for six expressions. Training on the AT&T dataset and testing on the Cohn-Kanade and JAFFE datasets (Unit: %).

	**Happiness**	**Sadness**	**Anger**	**Disgust**	**Surprise**	**Fear**
**Happiness**	**80**	6	4	2	3	5
**Sadness**	7	**79**	5	4	1	4
**Anger**	4	6	**77**	5	3	5
**Disgust**	5	5	8	**72**	4	6
**Surprise**	2	3	5	4	**83**	3
**Fear**	2	5	6	0	3	**84**
**Average**	**79.20**					

**Table 12.** Confusion matrix for the HL-FER (on the Cohn-Kanade dataset), while removing the preprocessing step (Unit: %).

	**Happiness**	**Sadness**	**Anger**	**Disgust**	**Surprise**	**Fear**
**Happiness**	**92**	8	0	0	0	0
**Sadness**	6	**94**	0	0	0	0
**Anger**	0	0	**95**	0	0	5
**Disgust**	0	0	0	**93**	7	0
**Surprise**	0	0	0	10	**90**	0
**Fear**	0	0	6	0	0	**94**
**Average**	**93.00**					

**Table 13.** Confusion matrix for the HL-FER (on the Cohn-Kanade dataset), while removing the hierarchical recognition step (Unit: %).

	**Happiness**	**Sadness**	**Anger**	**Disgust**	**Surprise**	**Fear**
**Happiness**	**89**	2	0	0	4	5
**Sadness**	0	**92**	4	4	0	0
**Anger**	0	5	**90**	5	0	0
**Disgust**	0	0	11	**89**	0	0
**Surprise**	4	0	0	6	**90**	0
**Fear**	0	2	9	0	0	**89**
**Average**	**89.80**					

**Table 14.** Confusion matrix for the HL-FER (on the JAFFE dataset), while removing the preprocessing step (Unit: %).

	**Happiness**	**Sadness**	**Anger**	**Disgust**	**Surprise**	**Fear**
**Happiness**	**91**	9	0	0	0	0
**Sadness**	7	**93**	0	0	0	0
**Anger**	0	0	**96**	0	0	4
**Disgust**	0	0	0	**90**	10	0
**Surprise**	0	0	0	8	**92**	0
**Fear**	0	0	8	0	0	**92**
**Average**	**92.33**					

**Table 15.** Confusion matrix for the HL-FER (on the JAFFE dataset), while removing the hierarchical recognition step (Unit: %).

	**Happiness**	**Sadness**	**Anger**	**Disgust**	**Surprise**	**Fear**
**Happiness**	**82**	5	3	2	5	3
**Sadness**	0	**90**	3	2	3	2
**Anger**	1	3	**93**	2	0	1
**Disgust**	4	0	6	**87**	0	3
**Surprise**	2	4	0	0	**88**	6
**Fear**	2	0	6	7	0	**85**
**Average**	**87.50**					

**Table 16.** Confusion matrix for the HL-FER (on the AT&T dataset), while removing the preprocessing step (Unit: %).

	**Happiness**	**Sadness**	**Anger**	**Disgust**	**Surprise**	**Fear**
**Happiness**	**89**	11	0	0	0	0
**Sadness**	9	**91**	0	0	0	0
**Anger**	0	0	**96**	0	0	4
**Disgust**	0	0	0	**90**	10	0
**Surprise**	0	0	0	12	**88**	0
**Fear**	0	0	10	0	0	**90**
**Average**	**90.66**					

**Table 17.** Confusion matrix for the HL-FER (on the AT&T dataset), while removing the hierarchical recognition step (Unit: %).

	**Happiness**	**Sadness**	**Anger**	**Disgust**	**Surprise**	**Fear**
**Happiness**	**92**	0	3	2	0	3
**Sadness**	3	**89**	0	2	3	3
**Anger**	2	4	**88**	0	2	4
**Disgust**	5	2	4	**85**	2	2
**Surprise**	2	3	0	4	**91**	0
**Fear**	1	4	0	8	3	**84**
**Average**	**88.16**					

**Table 18.** Confusion matrix of the HL-FER trained on the Cohn-Kanade dataset and tested on the AT&T dataset (Unit: %).

	**Happiness**	**Sadness**	**Anger**	**Disgust**	**Surprise**	**Fear**
**Happiness**	**91**	4	2	2	1	0
**Sadness**	4	**90**	3	0	0	3
**Anger**	1	2	**92**	0	0	5
**Disgust**	3	1	3	**89**	4	0
**Surprise**	0	2	3	3	**90**	2
**Fear**	0	2	3	1	2	**92**
**Average**	**90.70**					

**Table 19.** Confusion matrix of the HL-FER trained on the AT&T dataset and tested on the Cohn-Kanade dataset (Unit: %).

	**Happiness**	**Sadness**	**Anger**	**Disgust**	**Surprise**	**Fear**
**Happiness**	**89**	4	2	2	1	2
**Sadness**	2	**92**	0	2	4	0
**Anger**	0	3	**88**	4	2	3
**Disgust**	0	2	4	**90**	2	2
**Surprise**	5	1	2	3	**87**	2
**Fear**	1	3	1	2	3	**90**
**Average**	**89.30**					

**Table 20.** Comparison results of the HL-FER with some of the existing works for the three standard Cohn-Kanade, JAFFE, and AT&T datasets for six basic facial expression under the same settings as described in Section 3.1 (Unit: %).

**Existing Work**	[64]	[34]	[35]	[36]	[37]	[38]	[39]	[40]	[79]	**HL-FER**
**Average Accuracy Rate**	**91**	**86**	**87**	**85**	**81**	**69**	**82**	**86**	**87**	**98**

## Appendix

This sections provides an overview of the results of different other experiments performed during the course of the development of this work.

**Table A1.** Confusion matrix of PCA and HMM using Cohn-Kanade (CK), JAFFE (JA), and AT&T (AT) databases of facial expressions (Unit: %).

	**Happiness**			**Sadness**			**Anger**			**Disgust**			**Surprise**			**Fear**		
	**CK**	**JA**	**AT**	**CK**	**JA**	**AT**	**CK**	**JA**	**AT**	**CK**	**JA**	**AT**	**CK**	**JA**	**AT**	**CK**	**JA**	**AT**
**Happiness**	**63.5**	**55**	**50.5**	5.5	9.9	15.5	6.5	7.3	6.3	5.3	8.5	9.1	9.2	11	9.7	10	8.3	8.9
**Sadness**	6.3	13	21	**55.2**	**50.3**	**45**	10.5	6.9	8.3	9.4	12	9.9	5.9	8.9	7.7	12.7	8.9	8.1
**Anger**	4.5	6.7	8.9	12.5	9.8	10.9	**53.9**	**48**	**51.8**	11.9	10.7	9.5	6.3	9.8	8.9	10.9	15	10
**Disgust**	4.5	6.8	9.4	8.3	7.8	10.8	9.5	8.9	12	**65.8**	**55.8**	**40**	7.7	14	21	4.2	6.7	6.8
**Surprise**	8.9	8.5	8	6.9	5.6	7.9	8.6	8.7	8.9	9.8	10	11.6	**60.1**	**60**	**55.9**	5.7	7.2	7.7
**Fear**	9.6	6.7	7.8	11.8	10.7	9.1	10.5	13	15	9.7	8.4	8.4	8.5	8.2	12	**49.9**	**53**	**47.7**
**Average**	**56.33**			**50.16**			**51.23**			**53.85**			**58.66**			**50.2**		

**Table A2.** Confusion matrix of PCA + LDA and HMM using Cohn-Kanade (CK), JAFFE (JA), and AT&T (AT) databases of facial expressions (Unit: %).

	**Happiness**			**Sadness**			**Anger**			**Disgust**			**Surprise**			**Fear**		
	**CK**	**JA**	**AT**	**CK**	**JA**	**AT**	**CK**	**JA**	**AT**	**CK**	**JA**	**AT**	**CK**	**JA**	**AT**	**CK**	**JA**	**AT**
**Happiness**	**68.1**	**70.9**	**69.1**	7.9	8	9.1	5.6	5.4	6.2	7.9	5.9	5.5	4.7	3.6	5.8	5.8	6.2	4.3
**Sadness**	6.8	8.8	10	**72.3**	**69.5**	**65.5**	7.2	6	4.8	5.8	4.8	7.9	4	5	5.8	3.9	5.9	6
**Anger**	4.9	3.2	3.3	7.9	4.8	5.1	**70.9**	**74.9**	**75**	5.8	5.5	3.4	5.6	4.6	5.2	4.9	7	8
**Disgust**	3.9	6.7	4.3	8.5	7.9	7.5	7.8	7.8	6.5	**68.5**	**61**	**66.7**	5.6	10	8.9	5.7	6.6	6.1
**Surprise**	6.1	3.3	5.7	4.3	4.2	3.2	5.2	3.4	6.8	6.1	8.1	9	**73.6**	**78.9**	**71**	4.7	2.1	4.3
**Fear**	5.8	3.9	3.6	6.6	5.7	6.1	6.9	7.9	8.1	5.8	5.7	4.4	5.9	5.8	4.8	**69**	**71**	**73**
**Average**	**69.36**			**69.10**			**74.24**			**65.40**			**74.50**			**71.00**		

**Table A3.** Confusion matrix of ICA and HMM using Cohn-Kanade (CK), JAFFE (JA), and AT&T (AT) databases of facial expressions (Unit: %).

	**Happiness**			**Sadness**			**Anger**			**Disgust**			**Surprise**			**Fear**		
	**CK**	**JA**	**AT**	**CK**	**JA**	**AT**	**CK**	**JA**	**AT**	**CK**	**JA**	**AT**	**CK**	**JA**	**AT**	**CK**	**JA**	**AT**
**Happiness**	**69.2**	**65.7**	**63**	6.2	9.5	10	5.4	4.6	6.8	5.1	6.9	7.9	6.0	5.7	5.9	8.1	7.6	6.4
**Sadness**	2.8	9.6	9	**71.3**	**69**	**71.7**	8.6	3.7	5.1	5.8	4.8	3.8	7.7	6.9	6.1	3.8	6	4.3
**Anger**	4.7	3.6	4.3	8.7	4.9	5.6	**73.9**	**77.4**	**73.9**	5.9	3.8	4.6	0	4.3	3.7	6.8	6	7.9
**Disgust**	4.9	4	3.7	8.7	6	4.9	7.9	6.2	5.5	**65.8**	**71.5**	**69.4**	6.8	7.7	9.9	5.9	4.6	6.6
**Surprise**	3.2	5	3.6	4.7	5.9	3.4	5.3	6.4	6.1	3.9	8.9	9	**77.1**	**69**	**75**	5.8	4.8	2.9
**Fear**	5.8	2.6	4	8.7	3.8	4.1	7.9	8	7.6	6.5	4.5	5.3	6.7	5.3	6.2	**64.4**	**75.8**	**72.8**
**Average**	**65.96**			**70.66**			**75.06**			**68.90**			**73.70**			**71.00**		

**Table A4.** Confusion matrix of ICA + LDA and HMM using Cohn-Kanade (CK), JAFFE (JA), and AT&T (AT) databases of facial expressions (Unit: %).

	**Happiness**			**Sadness**			**Anger**			**Disgust**			**Surprise**			**Fear**		
	**CK**	**JA**	**AT**	**CK**	**JA**	**AT**	**CK**	**JA**	**AT**	**CK**	**JA**	**AT**	**CK**	**JA**	**AT**	**CK**	**JA**	**AT**
**Happiness**	**73.0**	**75.7**	**70.8**	5.8	7.2	11	5.8	4.7	0	6.9	0	6.2	0	5.2	7.2	8.5	7.2	4.8
**Sadness**	4.2	6.9	7.1	**75.1**	**73**	**75**	6.7	5.2	4.2	6.2	5.9	6	2.5	4.3	5	5.3	4.7	2.7
**Anger**	3	4.6	5.2	8.9	5.2	3.3	**69.9**	**71.2**	**76**	6.7	4.8	4.8	6.7	8	5.4	4.8	6.2	5.3
**Disgust**	0	6.7	5.5	6.6	0	6.4	9.2	8.4	7.2	**75.8**	**69.9**	**72.8**	8.4	9	8.1	0	6	0
**Surprise**	6.5	3	3.2	4.8	3.2	2.6	3.5	3.8	3.1	0	6	7.3	**79.3**	**80**	**81.4**	5.9	4	2.4
**Fear**	4.5	0	0	7.8	0	3	5.6	8.9	10	4.9	6.6	7	0	7.8	3	**77.2**	**76.7**	**77**
**Average**	**73.16**			**74.36**			**72.36**			**72.83**			**84.4**			**76.96**		

**Table A5.** Confusion matrix for the HL-FER using Yale B dataset of facial expressions (Unit: %).

	**Happiness**	**Sadness**	**Anger**	**Disgust**	**Surprise**	**Fear**
**Happiness**	**99**	1	0	0	0	0
**Sadness**	3	**97**	0	0	0	0
**Anger**	0	0	**98**	0	0	2
**Disgust**	0	0	0	**99**	1	0
**Surprise**	0	0	0	3	**97**	0
**Fear**	0	0	2	0	0	**98**
**Average**	**98.00**					
